# Liver ERα regulates AgRP neuronal activity in the arcuate nucleus of female mice

**DOI:** 10.1038/s41598-017-01393-0

**Published:** 2017-04-26

**Authors:** Valeria Benedusi, Sara Della Torre, Nico Mitro, Donatella Caruso, Alessandra Oberto, Claire Tronel, Clara Meda, Adriana Maggi

**Affiliations:** 1 Department of Pharmacological and Biomolecular Sciences, University of Milan, Via Balzaretti 9, 20133 Milan, Italy; 2 Center of Excellence on Neurodegenerative Diseases, University of Milan, Via Balzaretti 9, 20133 Milan, Italy; 3 UMR INSERM 930, Université François Rabelais de Tours, Tours, France; 4 Neuroscience Institute Cavalieri-Ottolenghi, University of Turin, NIT, Dept of Neuroscience, Regione Gonzole 10, 10043 Orbassano (To), Italy

## Abstract

Recent work revealed the major role played by liver Estrogen Receptor α (ERα) in the regulation of metabolic and reproductive functions. By using mutant mice with liver-specific ablation of *Erα*, we here demonstrate that the hepatic ERα is essential for the modulation of the activity of Agouti Related Protein (AgRP) neurons in relation to the reproductive cycle and diet. Our results suggest that the alterations of hepatic lipid metabolism due to the lack of liver ERα activity are responsible for a neuroinflammatory status that induces refractoriness of AgRP neurons to reproductive and dietary stimuli. The study therefore points to the liver ERα as a necessary sensor for the coordination of systemic energy metabolism and reproductive functions.

## Introduction

It is well known that, in the animal kingdom, metabolic and reproductive functions are strictly associated, principally in female organisms. In mammals, specific brain nuclei located in the hypothalamic region play a major role in such integration. In particular, the neurons expressing the orexigenic peptide AgRP in the arcuate nucleus of the hypothalamus (ARC) appear to represent an important crossroad for the selective detection and regulation of nutritional and reproductive functions, in virtue of their ability to regulate appetite and energy metabolism in response to estrogens and to interact with other neuronal systems (such as the Kisspeptin (Kiss1) secreting neurons)^1–3^ that regulate the secretion of the gonadotropin-releasing hormone (GnRH)^4^.

The ERα is highly expressed in this nucleus and estrogens are known to play a major modulatory action in the secretion of factors regulating female energy metabolism and reproductive functions^5–7^.

On the other hand, it is now becoming evident that, all along the course of phylogenesis, the liver (or the organs carrying out its function in less evolved animals) has been a key element for the integration of reproductive and metabolic activities. In fact, in oviparous and viviparous animals, nutritionally favorable conditions are indispensable for the synthesis of the liver proteins necessary for the maturation of the egg^8^. In mammals, liver appears to have amplified its cross-relation with the reproductive system by acquiring the capability to adapt its metabolism to the alternating necessities of the numerous phases of reproduction (ovulation, pregnancy, lactation)^8^. Liver ERα was suggested to act as the sensor of the changes occurring during reproduction and a regulator of reproduction-associated metabolism. Indeed, in the course of the different reproductive stages, the transcriptional activity of liver ERα is regulated, via unliganded and liganded activation, by dietary aminoacids and circulating estrogens^9, 10^ and this results in significant changes of lipid metabolism and transport^10–15^ and of the synthesis of the factors indispensable for the progression of the reproductive cycle (e.g. Insulin-like growth Factor-1, IGF-1)^9, 10, 16^. Thus, in view of their paired activity, a functional interconnection between the liver and AgRP neurons is conceivable, recent studies demonstrated that AgRP neurons regulate nutrient partitioning in liver through neuronal projections to preganglionic structures^17, 18^, but still unknown is whether the liver is able to influence the activity of AgRP neurons, in spite of the fact that these ARC cells were reported to be very sensitive to signaling of other peripheral organs relevant for energy metabolism, such as pancreas, adipose tissue and others^19–26^.

Considering the role of ERα in the control of liver homeostasis in response to reproductive and environmental cues, the aim of the present study was to evaluate whether the activity of this receptor has any influence on the function of the hypothalamic neurons of the AgRP/Kiss1 circuit. We here show that the ablation of liver *Erα* is associated with changes in the activity of AgRP neurons, thus pointing to the existence of a liver-hypothalamic circuit potentially relevant for the mutual control of energy expenditure and reproduction in mammals.

## Results

### In LERKO mice the activity of AgRP neurons fails to be regulated by the estrous cycle

Studies carried out in several laboratories, including ours, showed that AgRP mRNA fluctuates in the course of the reproductive cycle^7, 27^. We therefore investigated the extent to which liver ERα ablation influenced AgRP synthesis. As the estrous cycle in mouse lasts only 4 days and the extent of circulating estrogens may vary significantly within a day, animals were euthanized following a precise protocol in which the phase of the cycle was established by vaginal smears between 9 and 10 a.m., and the animals were euthanized between 2 and 5 p.m.

**Figure 1:**
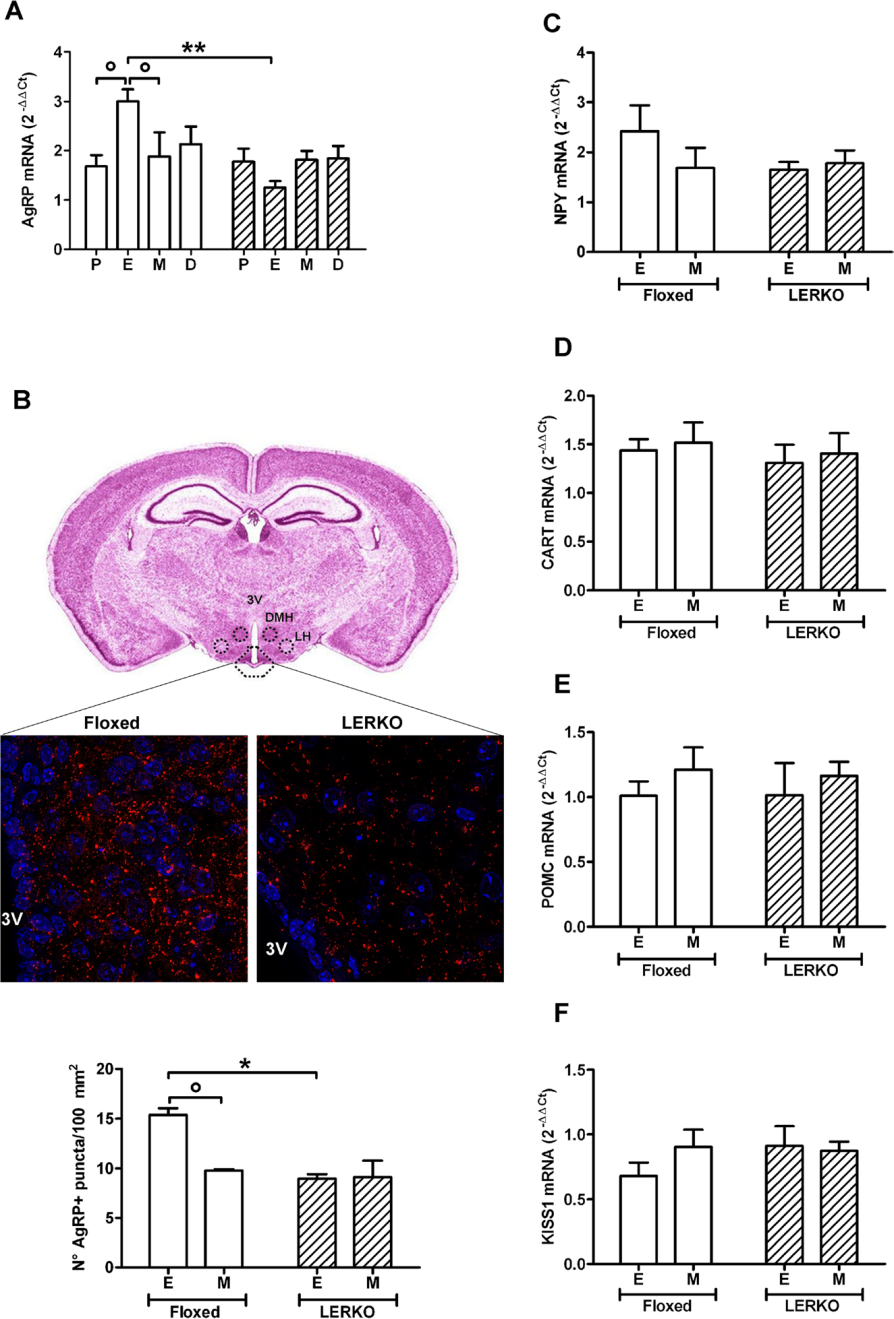
AgRP mRNA synthesis does not fluctuate during the estrous cycle in LERKO mice. AgRP mRNA measured by Real Time PCR in the hypothalamus of floxed and LERKO mice during the different phases of the estrous cycle, protestrus (P), estrous (E), metestrus (M) and diestrus (D) (**A**), representative images of AgRP staining in the arcuate nucleus of floxed and LERKO mice at E and quantification of AgRP positive synaptic puncta as evaluated by IHC for AgRP protein in the ARC of floxed and LERKO female mice at E (**B**), NPY mRNA (**C**), CART mRNA (**D**), POMC mRNA (**E**) and KISS1 mRNA (**F**) measured by Real Time PCR in the hypothalamus of floxed and LERKO female mice in E and M. 3 V = third ventricle. The image of the brain slice in Fig. 1B shows the areas used for the morphological analyses (from −1.70 mm to −1.58 mm from bregma) and is adapted from the web site: http://www.hms.harvard.edu/research/brain/atlas.html. The morphometric analysis was done as described in the methodology section. Data shown are the combined result of 2 separate experiments each with the following number of animals: rtPCR: n = 4 mice/exp for floxed, n = 8 mice/exp for LERKO. IHC: n = 3 mice/exp for floxed, n = 5 mice/exp for LERKO, Data are represented as mean ± SEM. Figure 1A: **p < 0.01, two-way ANOVA followed by Bonferroni post hoc test, p = 0.0236, DF = 1, F = 5.84, LERKO vs floxed, °P < 0.05, p = 0.5918, DF = 3, F = 0.65, P vs E vs M vs D, but the interaction between the two variables is significant (p = 0.0284). Figure 1B: *p < 0.05, two-way ANOVA followed by Bonferroni post hoc test, p = 0.0342, DF = 1, F = 6, LERKO vs FLOXED, °p < 0.05, unpaired two-tailed t-test, p = 0.0147, DF = 1, t = 8.161, E vs M. Figure 1C: p = 0.3076, DF = 1, F = 1.09, LERKO vs floxed, p = 0.3555, DF = 1, F = 0.89, Fig. 1D: p = 0.5608, DF = 1, F = 0.36, LERKO vs floxed, p = 0.6709, DF = 1, F = 0.19, E vs M, Fig. 1E: p = 0.9114, DF = 1, F = 0.01, LERKO vs floxed, p = 0.3957, DF = 1, F = 0.79, E vs M, Fig. 1F: p = 0.4722, DF = 1, F = 0.54, LERKO vs floxed, p = 0.5109, DF = 1, F = 0.45, E vs M.

Using the same experimental settings, prior experiments showed that in the whole hypothalamus the concentration of AgRP mRNA increased significantly in the course of estrous (E), while at proestrus (P), metestrus (M) and diestrus (D) was the same^27^. Consistent with the previous findings, Fig. 1A shows that in the hypothalamus of the floxed mice the content of the AgRP mRNA at E was the highest (+78% versus P, +59% versus M). In LERKO mice, AgRP mRNA content did not change in the course of the reproductive cycle (Fig. 1A). This could not be ascribed to a decreased expression of ERα in the hypothalamus, as demonstrated by the measurement of its mRNA during the cycle (Suppl. Fig. 1). To evaluate the extent to which the amount of AgRP mRNA reflected the activity of AgRP neurons, we carried out an IHC study (Representative images in Fig. 1B, upper panel) counting the number of terminals of neurons positive to AgRP immunostaining at E and M in the ARC (Fig. 1B, lower panel). In floxed female mice, the AgRP immunoreactivity at E was significantly higher than at M (+57%), in LERKO the immunoreactivity was as low as at M in the floxed counterpart and did not change at E supporting the previous results obtained in the whole hypothalamus (Fig. 1B, lower panel). No changes in AgRP immunoreactivity were observed in the paraventricular nucleus (PVN) of floxed and LERKO mice (Suppl. Fig. 2A).

We knew that the fluctuation of circulating estrogens is unaffected by the LERKO mutation^11^, thus the insensitivity of LERKO AgRP neurons to the alternation of the phases of the cycle could not be ascribed to alterations of circulating gonadal hormones, but had to be associated with factors dependent on liver ERα activity.

We next measured other hypothalamic mRNAs implicated in the control of energy metabolism. Neuropeptide Y (NPY) mRNA showed a trend to a fluctuation in floxed, but not in LERKO mice (Fig. 1C). Conversely, the anorexigenic cocaine-amphetamine-regulated transcript (CART) mRNA (Fig. 1D) and proopiomelanocortin (POMC) mRNA and protein (Fig. 1E and Suppl. Fig. 2B) did not show any measurable change associated with the phases of the cycle in both floxed and LERKO mice. Since it is known that the activity of the Kiss1 neurons is sensitive to estrogen^28^ and metabolic signals^29, 30^, we measured also Kiss1 mRNA content. As previously reported for animals in which liver ERα is expressed^27^, Kiss1 mRNA was the same at E and M in both floxed and LERKO mice (Fig. 1F), thus pointing to the fact that AgRP neurons are specifically affected by the progression of the cycle and by the absence of *Erα* expression in the liver.

### The refractoriness of LERKO AgRP neurons to the drop of circulating estrogens is associated with significant changes in microglia morphology

In spite of the lower content of orexigenic AgRP in the ARC, female LERKO mice are not leaner than their syngenic counterpart and show a comparable food intake (Suppl. Fig. 3), this was anticipated on the bases of prior reports showing that neonatal mutations affecting AgRP/NPY neuron signaling induce compensative responses that abolish the effect of the genetic alteration^17, 30–32^.

**Figure 2:**
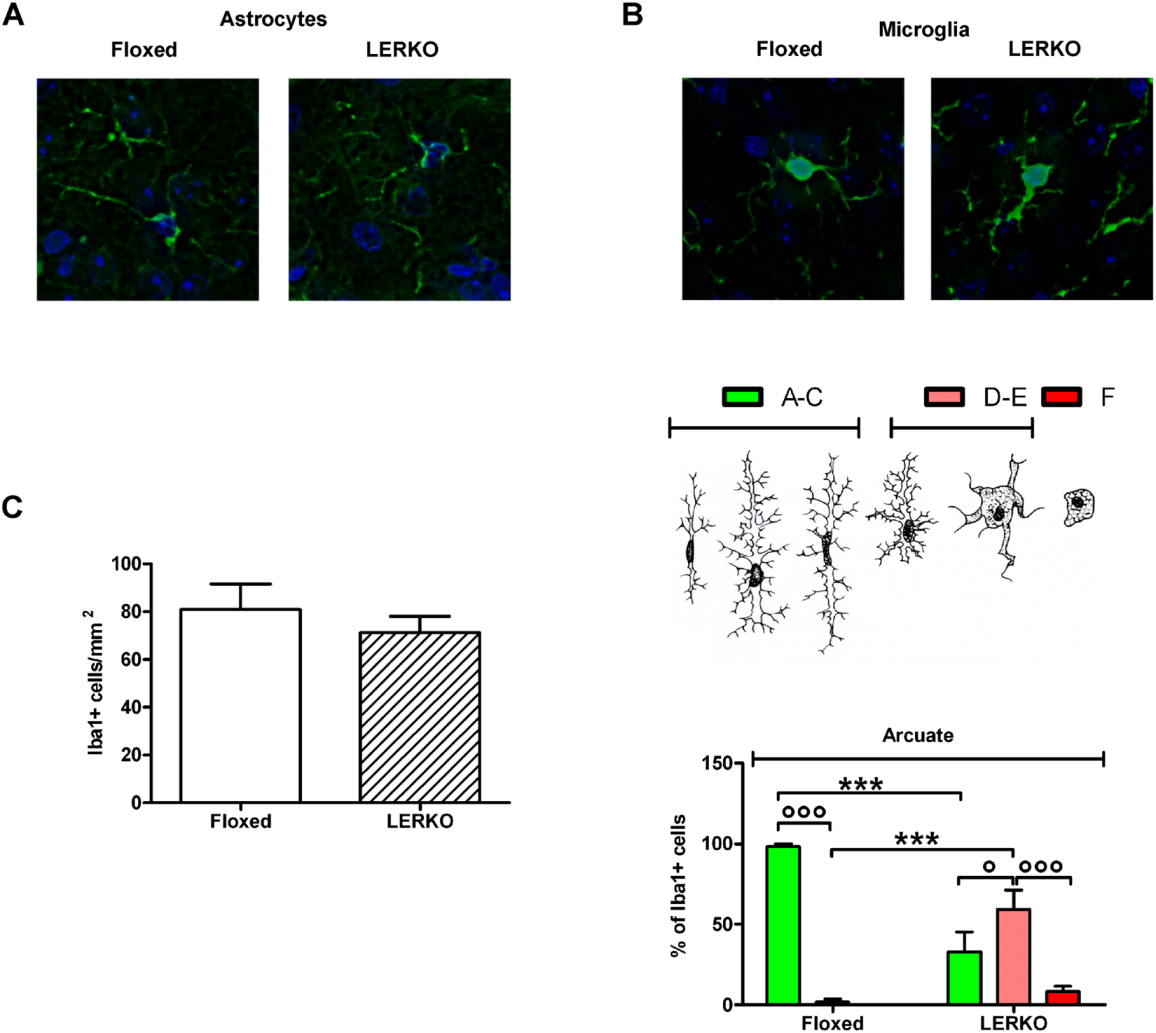
The absence of hepatic ERα affects the morphology of microglia, but not astrocytes. Representative images of astrocytes in the arcuate nucleus of floxed and LERKO mice stained with an anti-GFAP Ab (**A**), upper: representative images of microglia in the arcuate nucleus of FLOXED and LERKO mice at E stained with anti Iba1 Ab, middle: morphology of microglia at different stages of activation adapted from Kreutzberg *et al*., Trends is Neuroscience (1996)^83^, lower: percentages of microglia distribution across different phenotypes (**B**), percentage of Iba1-positive cells in the different stages of microglia activation in the arcuate nucleus of FLOXED and LERKO mice at E (**C**). Data shown are the combined result of 2 separate experiments (n = 5 mice/group/experiment). Data are represented as mean ± SEM. Figure 2B: °p < 0.05, °°°p < 0.001, ***p < 0.001, one-way ANOVA followed by Bonferroni post hoc test. p < 0.0001, DF = 2, F = 31.18 A–C vs D–E vs F, p = 1, DF = 1, F = 0 LERKO vs FLOXED, but the interaction between the two variables is significant (p < 0.0001). Figure 2C: p = 0.7855, DF = 17, t = 0.2765, two-tailed unpaired t-test.

On the other hand, several laboratories reported that the diet may induce alteration of the activity of AgRP/NPY neurons in association with local neuroinflammation^33, 34^: this effect is sexually dimorphic, as female mice appear to be less susceptible than males to such dietary effects and this insensitivity appears to be ERα-mediated^35^. This led us to investigate the state of neuroinflammation in the ARC of LERKO mice. We stained brains isolated from floxed and LERKO mice with antibodies against glial fibrillary protein (GFAP) and Ionized calcium binding adaptor molecule 1 (Iba1). The experiment was carried out in mice at E, as this is the phase in which we could best appreciate the unresponsiveness of LERKO AgRP neurons to the drop of circulating estrogens. The staining with the anti-GFAP antibody revealed that astrocytes were barely detectable in both floxed and LERKO and that their morphology was unaffected by the liver ablation of *Erα* (Fig. 2A). On the other hand, anti-Iba1 antibody immunoreaction showed that, in LERKO mice, microglia had a heterogeneous morphology and numerous cells had thicker and shorter branches (Fig. 2B, upper panel), indicative of the initial stages of conversion toward an overt inflammatory phenotype. It is in fact known that, in its surveilling status, microglia has thin and ramified branches that need to be retracted to enable the cell to acquire the globular phenotype characteristic of the response to an inflammatory stimulus (Fig. 2B, middle panel). Morphometric analysis of microglia showed that, in floxed mice, 98% of microglia was in the resting phenotype (A–C) and only 2% appeared to be in an initial stage of inflammation (D–E). This was expected, as these mice were females maintained in a standard diet. In LERKO mice 33% of the cells were resting (A–C), 59% showed an initial stage of activation (D–E), 8% were clearly activated (F) (Fig. 2B, lower panel). Further density analysis of these ARC cells showed that the number of cells was the same in floxed and LERKO mice (Fig. 2C, lower panel).

Thus microglia in LERKO appeared to be primed to activation. This finding was in line with previous studies associating neuroinflammation with an alteration of AgRP neuron activity^34, 36–38^. Interestingly, neuroinflammation was circumscribed to the brain area where the AgRP neurons are located, because no alterations in LERKO microglia morphology were observed in the dorsal-medium (DMH), lateral hypothalamus (LH), ventro-medial hypothalamus (VMH) and ventro-medial dorso-medial hypothalamus (VMDMH) (Suppl Fig. 4).

When we attempted to quantify the extent to which microglia priming was associated with the presence of inflammatory molecules, we failed to measure a significant increase in the hypothalamic content of mRNAs encoding molecules of the inflammatory cascade such as Macrophage Antigen Complex-1 (MAC1), CD11c, Interleukin-6 (IL6) and Macrophage Chemoattractant Protein-1 (MCP1) in the LERKO hypothalamus (Suppl. Fig. 5). We attributed this result to the fact that such a measurement was done in the entire hypothalamic region, while the area affected by the change was restricted to the ARC.

### High fat diet fails to activate microglia in LERKO mice

**Figure 3:**
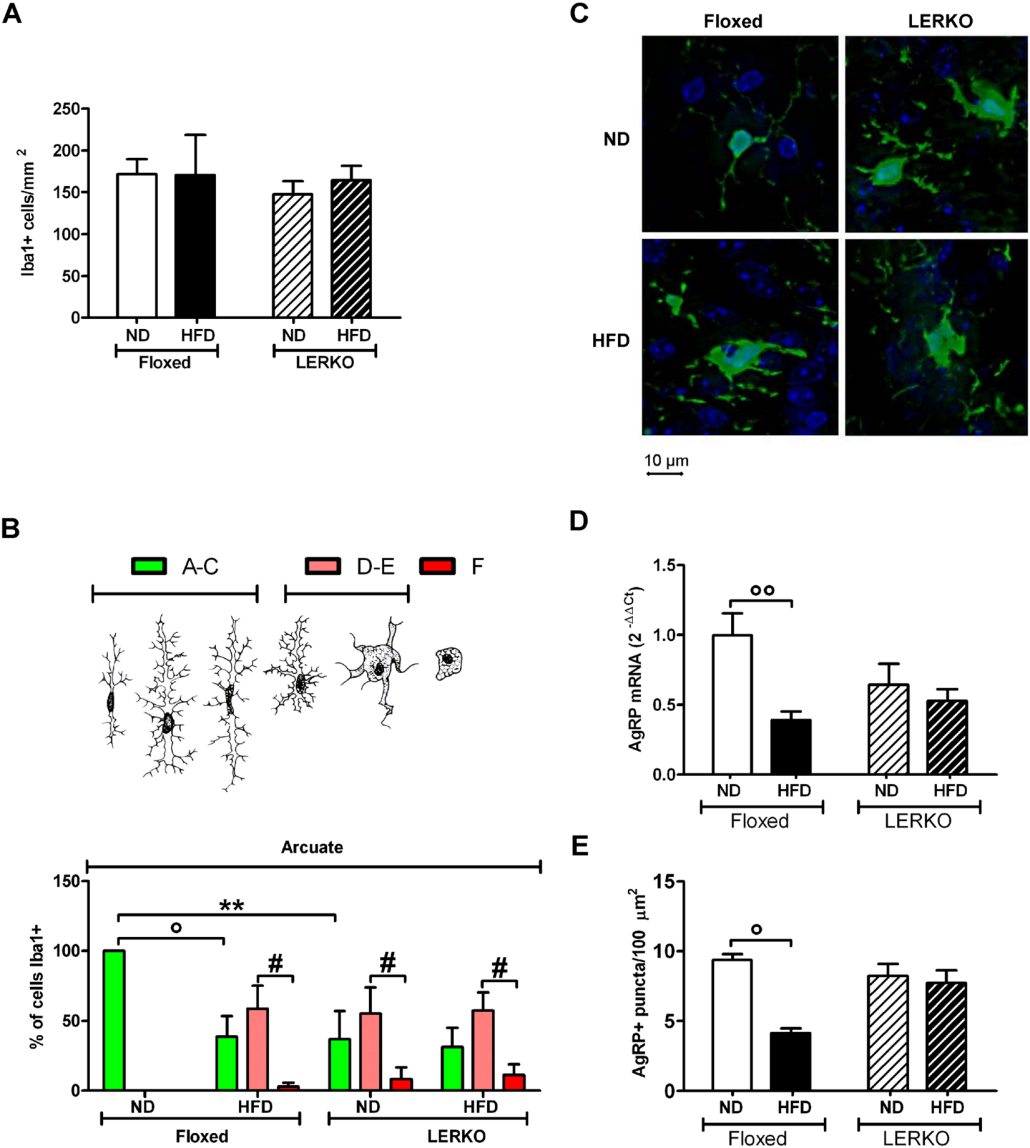
Neither microglia morphology in the ARC nor AgRP synthesis is affected by HFD in LERKO mice. Iba1 positive cells (**A**) percentages of microglia distribution across different phenotypes (**B**) in the arcuate nucleus of floxed and LERKO female mice in M after 4 months of ND or HFD, representative images of microglia stained with an antibody against Iba1 in the arcuate nucleus of the hypothalamus of floxed and LERKO female mice in metestrus after 4 months of ND or HFD (**C**), AgRP mRNA measured by Real Time PCR in the hypothalamus (**D**) and AgRP positive synaptic puncta, as evaluated by IHC for AgRP protein, in the arcuate nucleus of the hypothalamus (**E**) of floxed and LERKO female mice in M, after 4 weeks of ND and HFD. Data shown is combined results of 2 separate experiments with the following n: rtPCR: N = 5 mice/group/exp. IHC: N = 5 mice/group/exp. Data are represented as mean ± SEM. Figure 3A: two-way ANOVA followed by Bonferroni post hoc test, DF = 1, F = 0.11, p = 0.7475, HFD vs ND, DF = 1, F = 0.41, p = 0.5329, LERKO vs Floxed. Figure 3B: ^°, #^p < 0.05, *p < 0.01, two-way ANOVA followed by Bonferroni post hoc test, DF = 2, F = 12.75, p < 0.0001 A–C vs D–E vs F, DF = 3, F = 0, p = 1, LERKO vs SYN in each diet., Fig. 3D: °°p < 0.01, two-way ANOVA followed by Bonferroni post hoc test, DF = 1, F = 10.12, p = 0.0032 HFD vs ND, DF = 1, F = 0.89, p = 0.3512 LERKO vs Floxed. Figure 3E: °p < 0.05, two-way ANOVA followed by Bonferroni post hoc test, DF = 1, F = 10.14, p = 0.0058, HFD vs ND, DF = 1, f = 1.86, P = 0.1918, LERKO vs Floxed.

The finding of a primed microglia in the ARC of LERKO animals led us to hypothesize that, in these mutant mice, ARC microglia was more susceptible to inflammatory stimuli than floxed mice microglia. We tested this by exposing floxed and LERKO mice to HFD (60% Kcal derived from fat) for a period known to induce neuroinflammation (4 months). As HFD leads to a decrease of AgRP synthesis and increased inflammation^39–43^, in this experiment we devised to euthanize the mice at M because in this phase the activity of AgRP neurons was equally low in the two genotypes and this would have allowed us to better distinguish the diet-induced effect. Figure 3A shows that, in both genotypes, HFD did not modify the number of Iba1 positive cells. In floxed mice, the exposure to HFD increased significantly the number of cells with a less ramified phenotype (100% A–C in ND vs 38.7% A–C, 58.6% D–E, 2.8% F in HFD), this distribution of phenotypes was similar to what observed in LERKO in normal diet (36.8% A–C, 55% D–E, 8.2% F) (Fig. 3B). Unexpectedly, the HFD did not cause noticeable changes in LERKO microglia (31.1% A–C, 57.5% D–E, 11.2% F) (Fig. 3B and C), suggesting that the lack of ERα activity in the liver blunted the ability of ARC cells to sense the presence of diet-induced inflammatory stimuli. Again, we did not observe a change of the mRNAs of the proinflammatory molecules MAC-1, CD11c, IL-6 and MCP-1 in the entire hypothalamus (Suppl. Fig. 6). When we measured AgRP mRNA and counted the AgRP immunostained puncta we found no statistically significant differences between floxed and LERKO mice (Fig. 3D and E) maintained with the standard diet, this is in line with what expected in animals at M. HFD reduced AgRP mRNA and AgRP positive synaptic puncta in floxed, but not in LERKO mice (Fig. 3D and E).

Altogether the data obtained suggested that liver ERα has a role in maintaining AgRP neuron responsiveness to hormonal and dietary stimuli.

### The ablation of liver *Erα* is associated with plasma enrichment in molecules activating microglia

Prior experiments demonstrated that liver *Erα* plays a major role in the control of liver lipid metabolism and that a lack of estrogen signaling in liver is associated with lipid accumulation^11^ and inflammation (manuscript in preparation). This led us to ask whether in LERKO mice the state of microglia inflammation in the ARC could be ascribed to an increased concentration of circulating inflammatory molecules. To test this hypothesis, we exposed microglia N9 cells to plasma isolated from floxed or LERKO mice and measured the content on mRNAs encoding inflammatory molecules.

**Figure 4:**
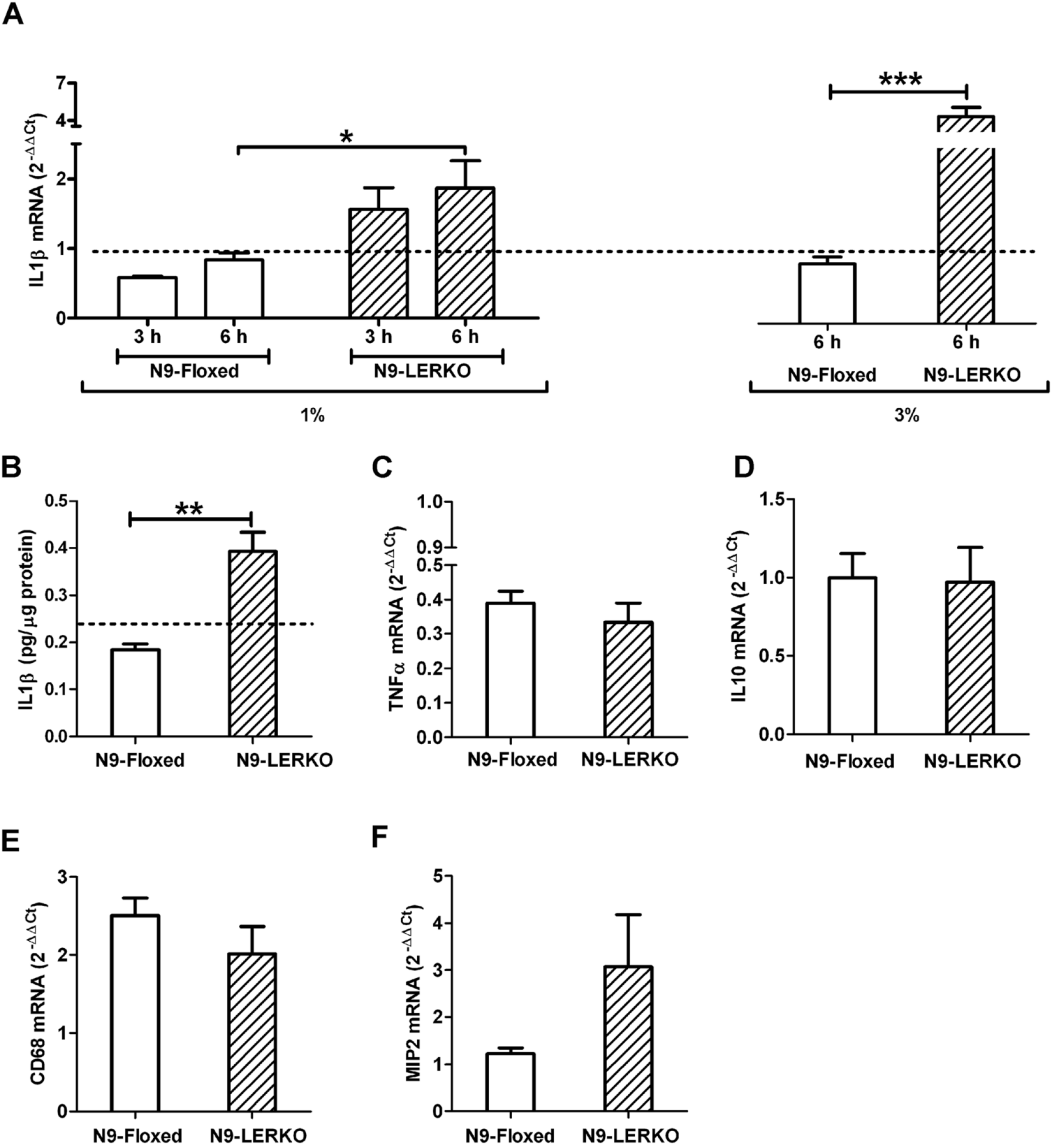
Plasma of LERKO mice induced IL1β production in N9 microglia cells while TNFα, MCP1, MIP2 and IL10 mRNAs are unaffected. IL1β mRNA measured by Real Time PCR in N9 cells treated with 1% or 3% of mouse plasma of floxed and LERKO mice in E for 3 and 6 hours. (**A**). IL1β protein content in cell lysate from N9 cells treated with 3% of mouse plasma of floxed and LERKO mice in E for 6 hours (**B**), TNFα mRNA (**C**), IL10 mRNA (**D**), CD68 mRNA (**E**), MIP2 mRNA (**F**) measured by Real Time PCR in N9 cells treated with 3% of mouse plasma of floxed and LERKO mice in E for 6 hours. The dotted line represents the value of untreated N9 cells. Data shown are the combined results of 2 separate experiments (Fig. 4A,C,D and F: n = 4 wells/group/exp, Fig. 4B: n = 2 wells/group/exp), all data are represented as mean ± SEM. The dotted lines represent the values of untreated cells. Figure 4A: *P < 0.05, one-way ANOVA followed by Bonferroni post hoc test, DF = 1, F = 11.10, p = 0.0088, N9-LERKO vs N9-SYN - treatment with 1% plasma, DF = 1, F = 0.85, p = 0.3795 3 h vs 6 h – treatment with 3% plasma, ***p < 0.0001, DF = 23, t = 5.646, two-tailed unpaired t-test, N9-LERKO vs N9-SYN – 3% treatment. Figure 4B: **p = 0.0027, DF = 5, t = 5.516, two-tailed unpaired t-test. Figure 4C–F: no significant difference was found between the two experimental groups by two-tailed unpaired t-test, Fig. 4C: p = 0.3899, DF = 23, t = 0.8764, Fig. 4D: p = 0.2541, DF = 18, t = 1.178, Fig. 4E: p = 0.2541, DF = 9, t = 1.178, Fig. 4F: p = 0.9218, DF = 23, t = 0.09930.

In the first experiment, we added to the medium of N9 cells the plasma isolated from the mice of the two genotypes at E to the final concentration of 1% or 3%. As shown in Fig. 4A, incubation for 3 and 6 hours with 1% plasma from LERKO, but not floxed mice, induced a significant increase of IL-1β mRNA content in N9 cells. This effect was magnified with the increase of the concentration of plasma from 1 to 3% (+154%) (Fig. 4A). Also at this higher concentration the plasma from floxed mice was without any measurable effect. The increased mRNA content reflected a significantly higher synthesis of intracellular IL-1β protein (Fig. 4B). These results indicate the presence of inflammatory molecules in the plasma of LERKO but not floxed mice. The treatment of N9 cells with 3% plasma from LERKO mice for 6 hours did not affect Tumor necrosis factor-α (*Tnf-α*), Interleukin-10 (*Il-10*) and *Cd68* mRNA expression, while Macrophage Inflammatory Protein-2 (MIP2) mRNA showed a trend to increase, still not significant (Fig. 4C–F).

Since we did not see any effect with heat inactivated plasma (Suppl. Fig. 7) and we knew that hepatic lipid synthesis in LERKO is highly affected by liver *Erα* ablation, we hypothesized that the inflammatory molecules in LERKO plasma were of lipid nature.

**Figure 5:**
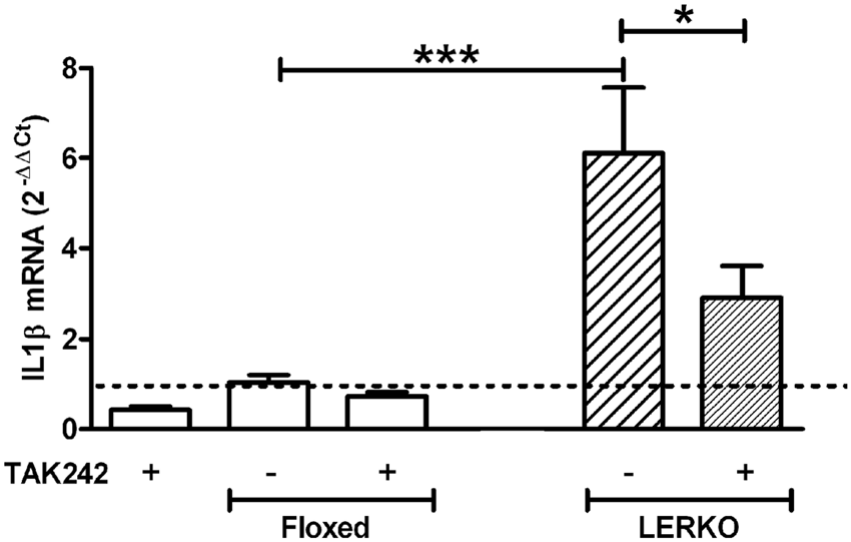
TLR4 is involved in the IL1β production induced in N9 cells treated with plasma of LERKO mice. IL1β mRNA measured by Real Time PCR in N9 cells treated with 3% of mouse plasma of floxed and LERKO mice in E for 6 hours with or without prior treatment with TLR4 inhibitor TAK242 10 μM 30 min before the plasma. Data shown is combined results of 2 separate experiments (N = 4 wells/group/exp). The dotted line represents the value of untreated N9 cells. All data are represented as mean ± SEM. *p < 0.05, ***p < 0.001, one-way ANOVA followed by Newman Keuls post hoc test, p = 0.0007, DF = 4, F = 9.794.

As lipids activate microglia through the TLR4 receptor^44^, we tested whether the exposure to the TLR4 inhibitor TAK242 prevented the activation induced by plasma from LERKO mice. Figure 5 shows that the response of N9 cells to LERKO plasma is blunted by the presence of the TLR-4 inhibitor, thus suggesting that the mediator/s responsible for microglia activation present in the LERKO plasma might be a lipid.

**Figure 6:**
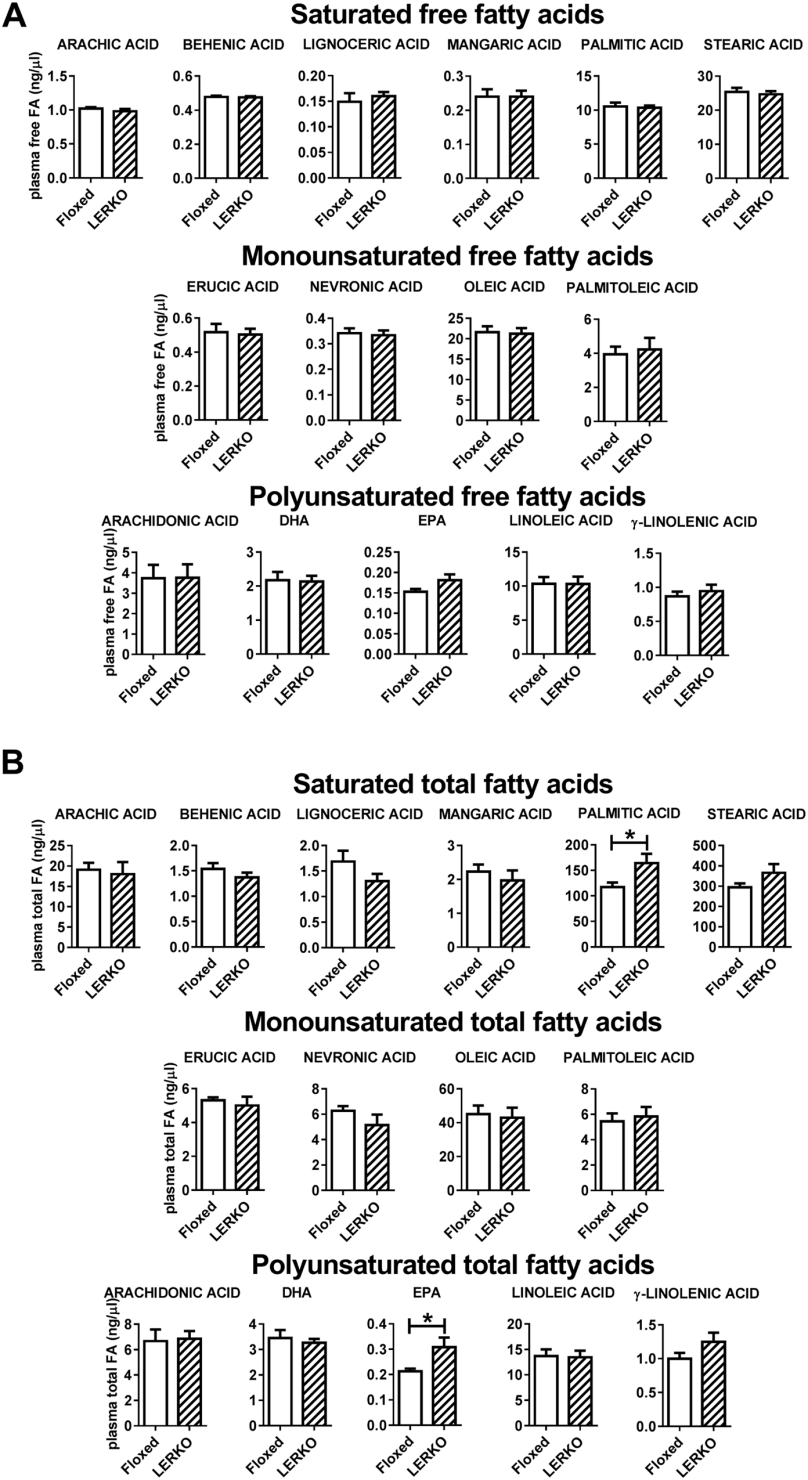
Total palmitic acid and EPA are increased in the plasma of LERKO mice. The content of free and total fatty acids indicated was measured in the plasma of floxed and LERKO mice in E by LC-MS/MS. Data shown is combined results of 2 separate experiments (N = 4–5 mice/exp). Statistical significance was assessed by two-tailed unpaired t-test. *p < 0.05, Fig. 6B: palmitic acid, p = 0.0327, DF = 11, t = 2.443, EPA, p = 0.0352, DF = 10, t = 2.434, EPA, p = 0.0352, DF = 10, t = 2.434.

This led us to measure free and total fatty acids in the plasma of floxed and LERKO mice in E by mass spectrometry. Plasma content of free fatty acids was unaffected (Fig. 6A) but total palmitic acid and eicosapentaenoic acid (EPA) were found to be significantly increased in LERKO mice (Fig. 6B). It is known that the saturated palmitic acid activates microglia and macrophages^45, 46^ through stimulation of TLR4 receptor^45^, thus suggesting its involvement in microglia activation in LERKO mice and in N9 cells *in vitro*.

## Discussion

The present study shows that the liver may play a role in the peripheral signaling to the arcuate nucleus and therefore that this organ may contribute to the overall ability of this Central Nervous System (CNS) nucleus to respond to dietary stimuli. Most interestingly, liver ERα is involved in this function. Estrogens are potent regulators of food intake and body weight in female mammals, including women. This effect is carried out mainly through a genomic action of the intracellular ERα, albeit some rapid, non-genomic actions have been also reported with the same receptor^47, 48^.

Still object of investigation is the extent to which estrogens are able to regulate the synthesis and secretion of the peripheral endocrine molecules that regulate the activity of the central nuclei for the metabolic control. What it is well described so far is that estrogens in the CNS increase the sensitivity to anorexigenic molecules such as leptin, released by adipocytes^49–52^, cholecystokinin (CCK), produced by small intestine^53, 54^ and insulin, synthesized by the pancreas^51, 52^ and, at the same time, decrease the response to the orexigenic peptide ghrelin from the gastro-intestinal tract^55, 56^.

In spite of its major role in energy metabolism, the liver ERα has never been reported to be involved in the regulation of the activity of the nuclei in the ARC relevant for energy homeostasis. Here, we demonstrate that the activity of AgRP neurons and of microglia in the ARC is altered following liver specific ablation of *Erα*. The molecules responsible for such signalling remain to be identified. Estrogens are known to induce the hepatic synthesis of IGF1^16^ and the circulating concentration of IGF1 is significantly lower in LERKO mice than in the floxed ones^9^: this, together with the fact that IGF1 does not affect the production of IL1β by N9 microglia (data not shown) would rule out any IGF1 participation in the estrogen-mediated signalling of liver energetic status to the central nuclei. On the other hand, we know also that liver ERα controls lipid metabolism^11^ and in the absence of liver ERα stimulation or in LERKO mice there is an increase of hepatic lipid synthesis^11, 57, 58^. Thus it could be hypothesized that in LERKO mice the augmented lipid deposits in liver and altered lipoprotein composition^11^ may determine the release in the circulation of a specific class of lipids able to activate microglia and influence the activity of AgRP neurons. Supporting the view of a lipid-based signalling are the following observations: (a) the phenomenon is restricted to the ARC, an area particularly exposed to fatty acid and lipid- associated molecules coming from the blood, also in virtue of the highly fenestrated blood brain barrier^59^, (b) when we treated N9 cells with heat-inactivated plasma from LERKO mice, we did not observe the microglia priming induced by plasma from LERKO mice (Suppl. Fig. 7C); (c) TLR4, known to be activated by fatty acids^60–63^, is necessary for IL1β production by microglia upon stimulation with the plasma of LERKO mice (d) selected fatty acids (a polyunsaturated fatty acid, EPA, and a saturated fatty acid, palmitic acid) are increased in the plasma of LERKO mice. This last observation may indicate a counterregulatory mechanism: indeed docosapentaenoic acid (DHA), a polyunsaturated fatty acid, has been able to tackle the proinflammatory effect of palmitic acid and LPS in different cellular models^64, 65^, EPA is a precursor of DHA^66, 67^, thus EPA accumulation may reflect a defect in DHA synthesis in LERKO mice. Furthermore, Valdearcos *et al*. demonstrated that microglia is a key mediator linking excess saturated fatty acids (SFAs) intake and hypothalamic inflammation, able to regulate neuronal stress and hypothalamic function in relation to excess dietary intake of saturated lipids^68^ and, most relevant for our findings, microglia restrains the responsiveness of ARC neurons to leptin when SFAs consumption is high^68^. This is in line with the refractoriness of AgRP neurons to the fluctuating levels of estrogen and to the HFD observed in LERKO mice, suggesting that the activated microglia in LERKO mice could be responsible for the altered function of AgRP neurons. We cannot rule out that, in LERKO mice, the lack of ERα influence on lipid metabolism may induce compensatory effects through vagal afferents described to influence the activity of arcuate nucleus^69, 70^.

The present study does not give a complete insight on the physiological role of the hepatic signalling to the ARC. The decreased activity of AgRP neurons here described in the LERKO mice did not affect body weight and food intake, previous studies showed that neonatal depletion of *Agrp* gene does not lead to anorexia while the acute-ablation does^32, 71–73^. It has been suggested that anorexia in models of acute deletion of *Agrp* in adult mice derives from hyperactivity of the parabrachial nucleus (PBN) due to the loss of the inhibitory GABAergic afferents from AgRP neurons^74–76^. Since AgRP afferents do not complete their neurocircuitry till 21 days of age, neonatal *Agrp* ablation does not result in PBN hyperactivity, probably due to lack of AgRP afferents in the PBN^77, 78^. Neonatal knockout of *Erα* in LERKO mice^79^ may lead to an early impairment of AgRP neuronal function, thus allowing the establishment of compensatory mechanisms.

Considering that the ARC controls food partitioning in liver and our prior studies pointing to the hepatic ERα as the sensor of nutrient availability necessary to regulate energy metabolism on reproductive functions^8^, it is tempting to speculate that the liver-ARC loop plays a role for the continuous monitoring and adaptation of reproductive functions upon nutrient availability. This would explain why in the absence the hepatic sensor of nutrient availability, ERα^9^, the LERKO ARC remains insensitive to the fluctuation of circulating estrogens and to HFD.

It is understandable that, during the course of evolution, reproductive functions shaped the mechanisms regulating energy metabolism in female mammals, creating an intricate network of interactions indispensable for the understanding of the loss of homeostasis of the energy metabolism occurring in women at the end of their fertile life. ERα is a clear central player in such mechanistic pathways, the hypothesis that we are putting forward is that liver ERα might have a unique function as a sensor of nutrient availability, driver of liver metabolism and reporter of liver functions to the CNS. Therefore its reduced activity after the menopause may represent a major driver for the obesity, inflammation and associated pathologies described for women in the post-menopausal period^80^. If this were the case, the present study provides a further indication that the correct replacement therapy for the post-menopause would be a Selective Estrogen Receptor modulator (SERM) able to target liver to re-establish the functions lost with the decrease of ovarian functions.

## Methods

### Animals

The floxed and LERKO mice were obtained and maintained as previously described^11^. Unless otherwise stated, the mice were 5–6 months of age at euthanasia. Vaginal smears were performed at 9:00–10:00 a.m. To avoid any possible confounding effect due to the circadian rhythm or feeding status, the mice were euthanized after 6 hours of fasting between 2:00 and 5:00 p.m.. The body weight and the food intake were calculated twice a week. The food intake was calculated as the amount of food consumed by the single cage averaged by the number of mice and the number of days, then the Kcal consumed were calculated for each diet. All animal experimentation was performed in accordance with the ARRIVE guidelines and the European guidelines for animal care and the use of experimental animals, approved by the Italian Ministry of Research and University, and controlled by a departmental panel of experts.

### Diets

The control diet (ND) consisted of 4.3% fat, 67.3% carbohydrate, and 19.2% protein with 10% Kcal derived from fat, 70% Kcal derived from carbohydrates and 20% Kcal derived from proteins (D12450B, Research Diets, NJ, USA). The HFD used in the experiments consisted of 35% fat, 26% carbohydrate, and 26% protein with 60% of Kcal derived from fat, 20% Kcal from carbohydrates and 20% Kcal from proteins (D12492, Research Diets, NJ, USA). Free access to the diets was provided. Mice were given ND and HFD from 2 to 6 months of age. They were euthanized by anaesthesia overdosing (Ketamine and xilazine) and cervical dislocation.

### Blood sampling procedures

Blood was collected by cardiac puncture under profound anaesthesia and before cervical dislocation. EDTA 12.5 mM was added to blood immediately to prevent clotting and plasma was obtained by centrifugation at 8000 rpm at 4 °C. Plasma was stored at −80 °C.

### Real-Time PCR Gene Expression Analysis

The hypothalami were homogenized in TRIzol® (Life Technologies, Carlsbad, CA), (6% w/v) using a TissueLyser (QIAGEN, Milan, Italy) while N9 cells were lysed with the RLT lysis provided by the RNeasy Mini Kit (QIAGEN, Milan, Italy) supplemented with 10% β-mercaptoethanol. RNA was purified using the RNeasy Mini Kit, according to the manufacturer’s instructions. cDNA synthesis, real-time PCR, and data analysis were performed as previously described^81^. Real time PCR analyses was performed on triplicates. The primers used for the rtPCR reactions were as follows: *AgRP* Mm00475829_g1, *Npy* (Mm03048253_m1), *Cart* (Mm04210469_m1), *POMC* (Mm00435874_m1), *Kiss1 (*Mm03058560_m1), *Esr1* Mm00433147_m1, *Il1β* Mm00434228_m1, *Tnfα* (Mm00443258_m1), *Il-10* (Mm00439614_m1), CD68 Mm03047340_m1, *Mip2* (Mm00436450_m1), Mac1 *(Mm00434455_m1), Cd11c* (Mm00498698_m1), *Il-6* (Mm00446190_m1), *Mcp1* (Mm00441242_m1). Primers for the *36b4* gene were used as reference (forward: 5′-GGCGACCTGGAAGTCCAACT-3′ and reverse: 5′-CCATCAGCACCACAGCCTTC-3′).

### Histological Analysis

For AgRP, GFAP and Iba 1 analysis, brains were taken and immersed in 4% buffered paraformaldehyde for 24 h and then paraffin embedded. Serial sections of paraffin-embedded brains were cut at 5 μm and used to perform different immunostainings. On selected sections, the following immunohistochemical stainings were carried out: Agouti Related Protein (AgRP rabbit polyclonal antibody, 1:200, Phoenix), Ionized calcium binding adaptor molecule 1 (Iba1 rabbit polyclonal antibody, 1: 500, Wako Chemicals) and glial fibrillary acidic protein (GFAP, mouse Alexa Fluor 488- conjugated monoclonal antibody, 1:1000, Chemicon). Nuclei were labeled with Hoechst 33342 (Sigma). For quantitative analysis, brain slices images (1300 × 1030 pixels) in the area of the arcuate nucleus (−1.70 mm to −1.58 mm from bregma) and of the paraventricular nucleus (−0.94 to −0.82 from bregma) were captured using the x40 objective of a Zeiss Axioscope microscope equipped with a digital camera (Carl Zeiss, Thornwood, NY).

For POMC analysis brains were taken and immersed in 4% buffered paraformaldehyde for 24 h, then they were cryoprotected in a solution of sucrose 30% in phosphate buffer 0.1 M. Brains were cut at 25 μm and the immunohistochemical staining for POMC was carried out (POMC rabbit polyclonal antibody, 1:5000, Phoenix). For quantitative analysis, brain slices images (2088 × 1550 pixels) in the area of the ARC were captured using the x10 objective of a Zeiss Axioplan microscope equipped with a digital camera (Leica DFC 320, Wetzlar, Germany).

#### Morphometric analysis of Iba1 stained microglia

3 images of each half of the arcuate nucleus from each of 3 different slices/mouse and 2 images of LH, DMH, VMDMH and VMH from each of three different slices/mouse were taken. The size of the fields analysed was 173 μm × 218 μm. 5 mice per group were analysed. Morphological analyses was performed in a double blind manner. ImageJ software was used to measure immunoreactivity through a threshold method and the number of positive pixels and the extension of area of interest were used to determine the fractional area covered by the specific signal.

#### Quantification of AgRP positive puncta

3 images of each half of the arcuate and paraventricular nucleus from each of 3 different slices/mouse were taken. The size of the fields analysed was 173 μm × 218 μm. 5 mice per group were analysed. The number of AgRP positive synaptic puncta was quantified by sharpening the contour with the software Axiovision 4.6.3 and then using the plugin Puncta Analyzer (written by Bary Wark, available upon request, c.eroglu@cellbio.duke.edu) under the ImageJ analysis software platform.

#### Quantification of POMC positive puncta

2 images of each half of the arcuate from each of 4 different slices/mouse were taken. 3 mice per group were analysed. ImageJ software was used to quantify the number of POMC positive synaptic puncta.

### Cell culture

The N9 microglia cells were developed by immortalizing mouse primary microglia cells with the v-myc or v-mil oncogenes of the avian retrovirus MH2^82^. This cell line displays several phenotypic traits of primary microglia mouse cells^75^. N9 cells were routinely maintained in Iscove’s Modified Dulbecco’s Medium (IMDM, Gibco by Life Technologies) supplemented with 1% antibiotic-antimycotic (Gibco by Life Technologies) and 10% Fetal Bovine Serum (Sigma-Aldrich).

For RNA extraction cells were plated at a density of 3.5 × 10^5^ cells/well in 6-well plates. After 16 hours medium was changed and cells were treated with plasma as indicated for 6 hours and then lysed. Plasma for treatment was obtained from floxed and LERKO mice as described above, cells were treated with pools from 6–8 mice.

For TLR-4 experiments, cells were treated with the TLR-4 inhibitor TAK-242 (Calbiochem) 10 μM 1 hour before the treatment with mouse plasma.

### Fatty acid profile by liquid chromatography-tandem mass spectrometry (LC-MS/MS)

20 μl of plasma from floxed and LERKO mice were added to 100 μl of MeOH:Acetonitrile 1:1 containing the internal standards (IS) [U-13C] linoleic acid (C18:2, Sigma Aldrich). Proteins were precipitated by centrifugation and the supernatant containing lipids was recovered. For the quantification of free fatty acids an aliquot of the supernatant was directly analyzed as described below.

Total fatty acids were obtained from samples by acid hydrolysis. Specifically, an aliquot of remaining supernatant was resuspended in equal volume of chloroform/MeOH 1:1 v/v. and of 1 M HCl:MeOH (1:1, v/v) and shaken for 2 h. Then, chloroform:water (1:1 v/v) was added (equal volume of the starting supernatant) and the lower organic phase was collected, transferred into a new tube and dried under nitrogen flow. The residue was then resuspended in MeOH.

For fatty acid quantification, aliquots of each sample were diluted 1:10 in MeOH/water (50:50 v/v), transferred into a 96 well-plate and placed in a HTS-CTC PAL auto-sampler for LC–MS/MS analysis. Quantitative analysis was performed with calibration curves prepared and analyzed daily by electrospray ionization (ESI) using an API 4000 triple quadrupole instrument (AB Sciex, USA). The LC mobile phases were: water/10 mM isopropylethylamine/15 mM acetic acid (phase A) and MeOH (phase B). The gradient (flow rate 0.5 ml/min) was as follows: T 0: 20% A, T 20: 1% A, T 25: 1% A, T 25.1: 20% A, T 30: 20% A. The Hypersil GOLD C8 column (100 mm × 3 mm, 3 μm) was maintained at 40 °C. The mass spectrometer was operated in selective ion monitoring (SIM)/SIM mode. Data were obtained using Multiquant software (AB Sciex, USA).

### Statistical analysis

Statistical Analyses—Data are represented as mean ± S.E.M and statistical significance was verified using GraphPad Prism 5 ® software (GraphPad Software, San Diego, CA). Statistical significance was assessed by two-tailed unpaired t test for comparisons between two groups, one-way or two-way ANOVA followed by Bonferroni or Newman-Keuls post hoc tests were used for comparisons of multiple groups.

## Electronic supplementary material

Supplementary information
