# Supplementary material for: Liver ERα regulates AgRP neuronal activity in the arcuate nucleus of female mice
Source: Sci Rep. 2017 Apr 26;7:1194. doi: 10.1038/s41598-017-01393-0 (PMC5430776; doi:10.1038/s41598-017-01393-0)

TITLE: Liver ERα regulates AgRP neuronal activity in the arcuate nucleus of female mice

AUTHORS: Valeria Benedusi, Sara Della Torre, Nico Mitro, Donatella Caruso, Alessandra Oberto, Claire Tronel, Clara Meda and Adriana Maggi*

SUPPLEMENTARY INFORMATION

Supplementary figure 1: ERα mRNA synthesis in the hypothalamus is not affected by the absence of hepatic ERα. ERα mRNA measured by Real Time PCR in the hypothalamus of floxed and LERKO mice at 5-6 months of age during the different phases of the estrous. Data shown is combined results of 2 separate assays (n=4/group/assay for mRNA). Data are represented as mean ± SEM. No significant differences were found between the experimental groups. Two-way ANOVA followed by Bonferroni post hoc test, p=0.8091, DF=1, F=0.06, LERKO vs floxed; p=0.0562, DF=3, F=2.92, P vs E vs M vs D.

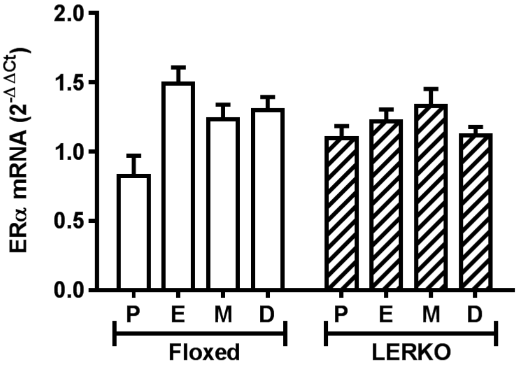

Supplementary figure 2: AgRP protein in the PVN and POMC protein in the ARC are not affected by the absence of hepatic ERα. Quantification of AgRP positive (A) and POMC positive (B) synaptic puncta as evaluated by IHC in the PVN for AgRP protein evaluation and in the ARC for POMC protein evaluation, of floxed and LERKO female mice at E (n=4-6/group for AgRP, n=3/group for POMC). Data are represented as mean ± SEM. No significant differences were found between the experimental groups: unpaired two-tailed t test, Suppl. Fig. 2A: p= 0.6865, DF= 8, t=0.4186, Suppl. Fig. 2B: p=0.1380, DF=4, t=1.85.

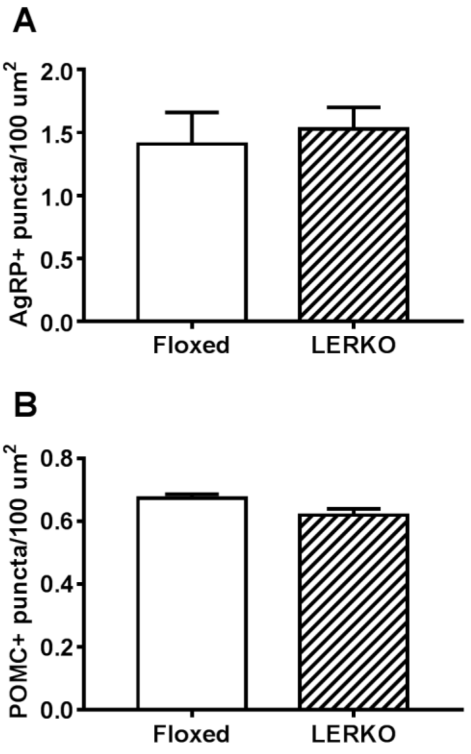

Supplementary figure 3: Body weight and food intake are not affected by the absence of hepatic ERα. Body weight (A) and mean daily food intake (B) of floxed and LERKO mice at 5-6 months of age. Data shown is combined results of 2 separate assays (n=8/group/assay for body weight and food intake). Data are represented as mean ± SEM. No significant differences were found between the experimental groups. Suppl. Fig. 3A and 3B, unpaired two-tailed t test, Suppl. Fig. 3A: p= 0.3265, DF= 16, t=1.012, Suppl. Fig. 3B: p=0.2429, DF=3, t=1.450.

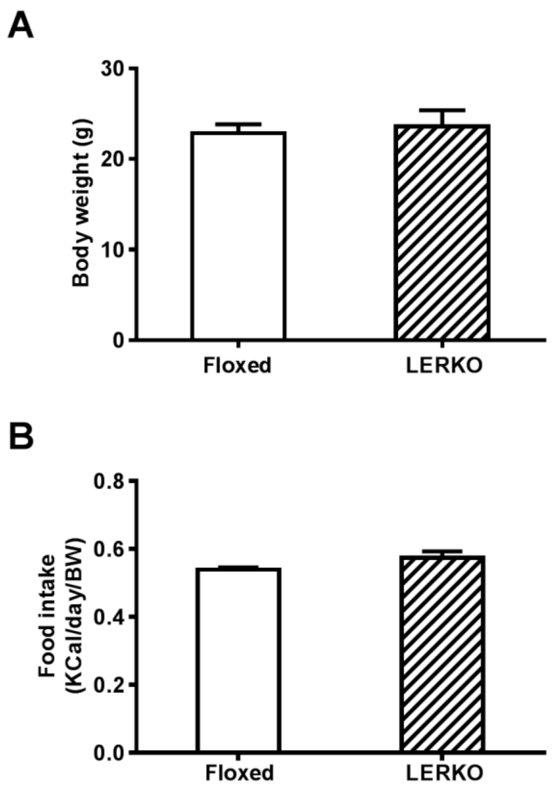

Supplementary figure 4: microglia morphology in the DMH, LH, VMDMH and VMH is not affected by the absence of hepatic ERα. Percentages of microglia distribution across different phenotypes in the DMH (A), LH (B), VMDMH (C) and VMH (D) Iba1-dependent fluorescence intensity (E) and Iba1 positive cells (F) in the DMH, LH, VMDMH and VMH of floxed and LERKO mice in M. Data shown is combined results of 2 separate assays (*n*= mice/group/assay). 6 fields/mouse were evaluated. Data are represented as mean ± SEM. °°°, p<0.001, one-way ANOVA followed by Bonferroni *post hoc* test, Suppl. Fig. 4A: p<0.0001, DF=2, F=637.98 A-C vs D-E vs F, p=1, DF=1, F=0, LERKO vs floxed in DMH, Suppl. Fig. 4B, C and D: replicate values are identical, so ANOVA is impossible; Suppl. Fig. 4E: : unpaired two-tailed t test, DMH, p=0.2051, DF=8, t=1.379; LH, p=0.1968, DF=8, t=1.408; VMDMH, p=0.1654, DF=8, t=1.636; VMH, p=0.2291, DF=8, t=1.302. Suppl. Fig. 4F: unpaired two-tailed t test, DMH, p=0.8030, DF=8, t=0.2579; LH, p=0.0543, DF=8, t=2.253; VMDMH, p=0.6692, DF=8, t=0.4435; VMH, p=0.8432, DF=8, t=0.2043.

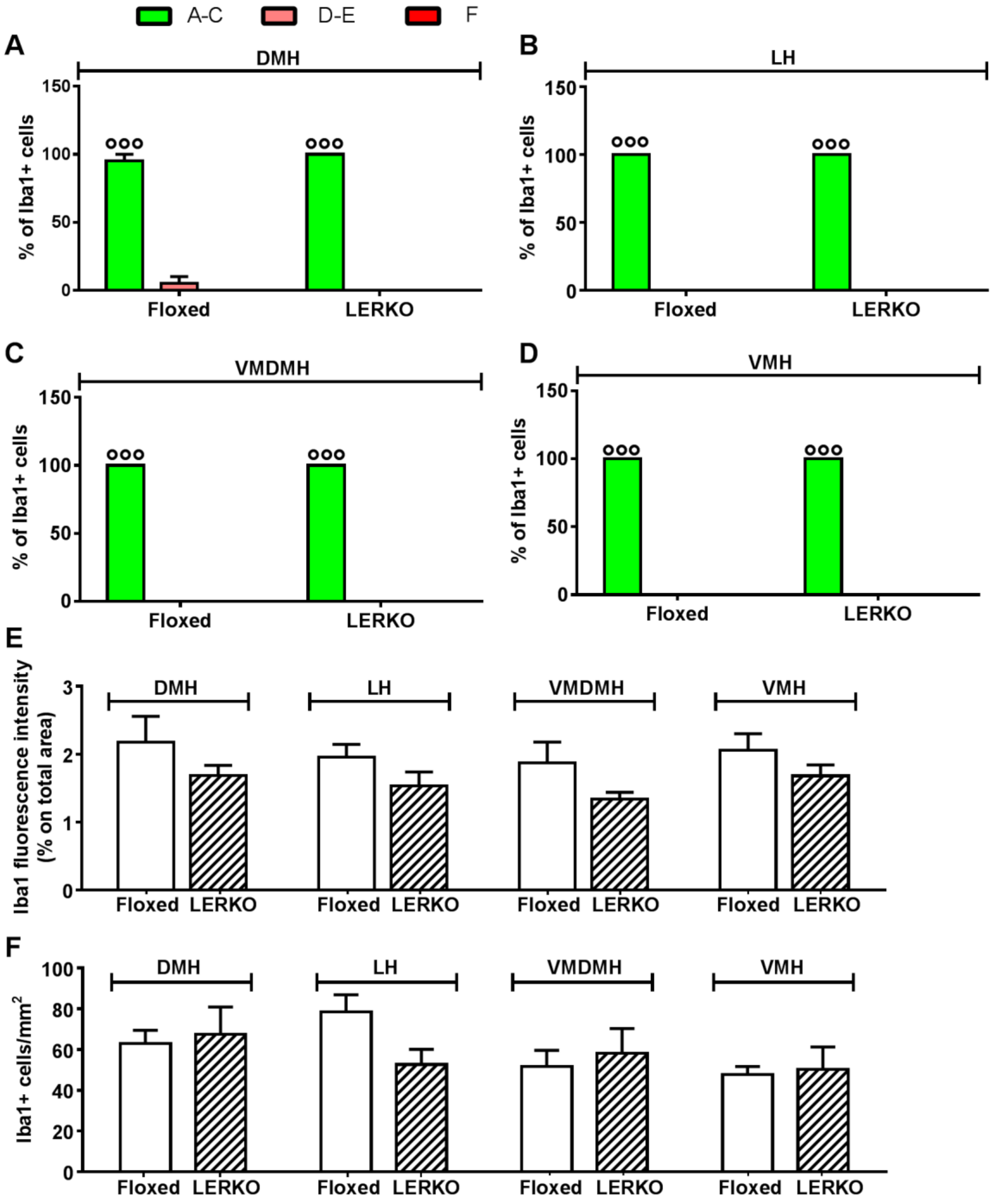
A-C
D-E
F
A
DMH
% of Iba1+ cells
150
100
50
0
Floxed
LERKO
B
LH
C
VMDMH
D
VMH
E
Iba1 fluorescence intensity (% on total area)
3
2
1
0
F
Iba1+ cells/mm2
100
80
60
40
20

Supplementary figure 5: the synthesis of mRNAs encoding for proinflammatory mediators in the entire hypothalamus is not affected by the absence of hepatic ERα. mRNA levels of MAC1 (A), CD11c (B), IL6 (C) and MCP1 (D) measured by Real Time PCR in the hypothalamus of floxed and LERKO female mice in E. Data shown is combined results of 2 separate assays (*n*=5 mice/group/assay). Data are represented as mean ± SEM. No significant difference was found between the two experimental groups by unpaired two-tailed t-test. Suppl. Fig. 5A: p=0.7208, t=0.3676, DF=10; Suppl. Fig. 5B: p=0.6306, t=0.4934, DF=12; Suppl. Fig. 5C : p=0.2172, t=1.302, DF=12; Suppl. Fig. 5D: p=0.7732, t=0.2961, DF=10.

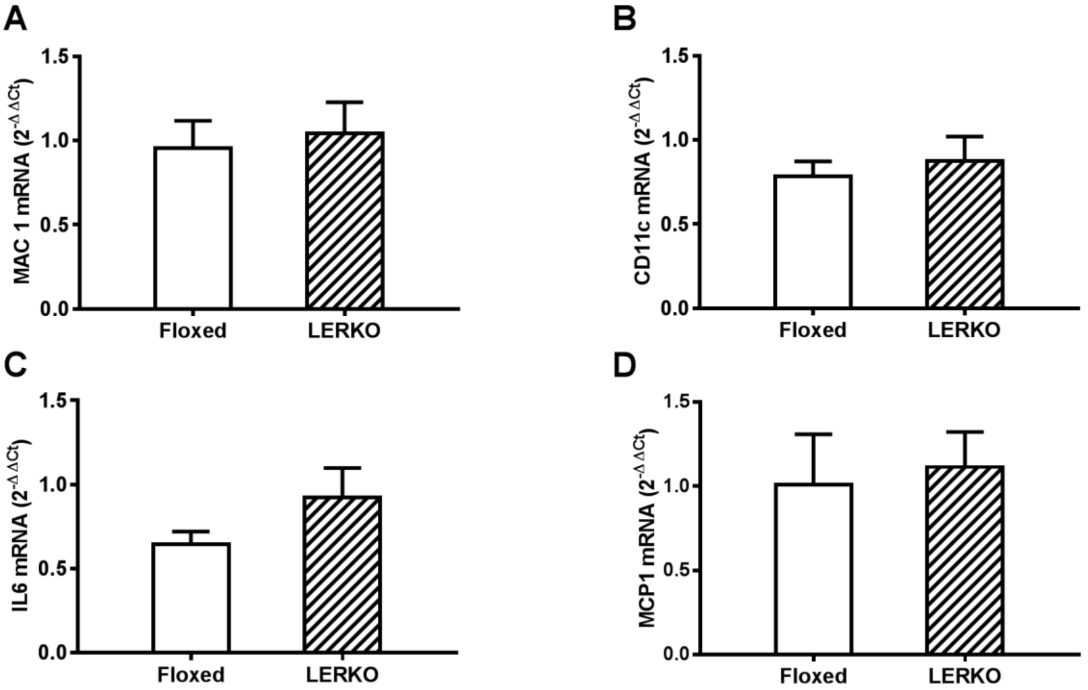

Supplementary figure 6: HFD does not affect the synthesis of mRNAs encoding for proinflammatory mediators in the entire hypothalamus in the presence or absence of hepatic ERα. mRNA levels of MAC1 (A), CD11c (B), IL6 (C) and MCP1 (D) measured by Real Time PCR in the hypothalamus of floxed and LERKO female mice in M, after 4 weeks of ND and HFD. Data shown is combined results of 2 separate assays (*n*=4 mice/group/assay). Data are represented as mean ± SEM. No significant difference was found between the experimental groups by two-way ANOVA followed by Bonferroni *post hoc* test. Suppl. Fig. 6A: p=0.3476, DF=1, F=0.91, HFD vs ND, p=0.1785, DF=1, F=1.88, LERKO vs floxed. Suppl. Fig. 6B: p=0.3782, DF=1, F=0.80, HFD vs ND, p=0.6009, DF=1, F=0.28, LERKO vs floxed. Suppl. Fig. 6C: p=0.4687, DF=1, F=0.54, HFD vs ND, p=0.4503, DF=1, F00.58, LERKO vs floxed. Suppl. Fig. 6D: p=0.3522, DF=1, F=0.89, HFD vs ND, p=0.0808, DF=1, F=3.24, LERKO vs floxed.

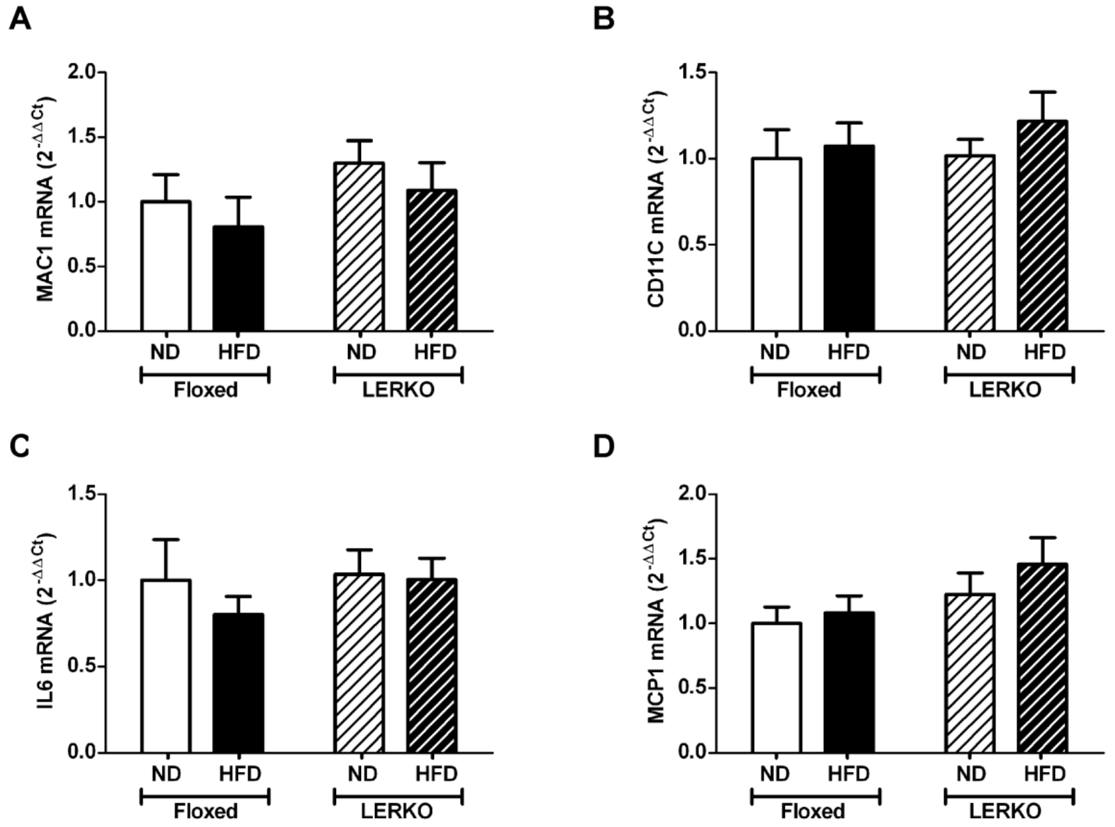

Supplementary figure 7: protein degradation by heat inactivation does not affect the increase in IL1β production induced by plasma from LERKO mice in N9 cells. IL1β mRNA measured by Real Time PCR in N9 cells treated with 3% of control or heat inactivated (HI) mouse plasma of floxed and LERKO mice in E for 6 hours. The dotted line represents the value of untreated N9 cells. Data shown is the result of a single assay (*n*=4 wells/group). Data are represented as mean ± SEM. ***, P<0.001, two-way ANOVA followed by Bonferroni *post hoc* test, DF=1, F=111.46, p<0.0001, N9-LERKO vs N9-floxed; DF=1, F=0.10, p=0.7602, HI vs CTRL.

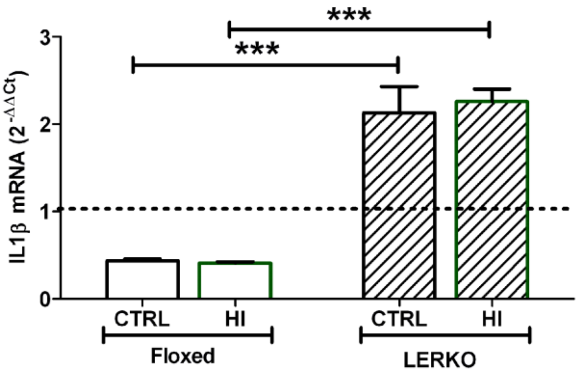

Supplement: Supplementary file 1 — Supplementary information [file 41598_2017_1393_MOESM1_ESM.pdf]
